# New Delhi Metallo-β-Lactamase in *Klebsiella pneumoniae* and *Escherichia coli,* Canada

**DOI:** 10.3201/eid1701.101358

**Published:** 2011-01

**Authors:** Michael R. Mulvey, Jennifer M. Grant, Katherine Plewes, Diane Roscoe, David A. Boyd

**Affiliations:** Author affiliations: Public Health Agency of Canada, Winnipeg, Manitoba, Canada (M.R. Mulvey, D.A. Boyd);; Vancouver General Hospital, Vancouver, British Columbia, Canada (J.M. Grant, K. Plewes, D. Roscoe)

**Keywords:** Multidrug resistance, New Delhi metallo-β-lactamase, Klebsiella pneumoniae, Escherichia coli, carbapenemase, bacteria, expedited, Canada, dispatch

## Abstract

Multidrug-resistant *Klebsiella pneumoniae* and *Escherichia coli* isolates harboring New Delhi metallo-β-lactamase (NDM-1) were isolated from a patient who had returned to Canada from India. The NDM-1 gene was found on closely related incompatibility group A/C type plasmids. The occurrence of NDM-1 in North America is a major public health concern.

Carbapenem-hydrolyzing β-lactamases, i.e., carbapenemases, in bacterial clinical isolates are an increasing concern because they often also confer resistance to most other β-lactam antimicrobial agents. Among *Enterobacteriaceae*, carbapenemases are mainly found in the Ambler class A penicillinase or class B metallo-enzyme groups. VIM and IMP are the most frequently acquired class B enzymes and are commonly found in southern Europe and the Far East, rarely in North America. *Klebsiella pneumoniae* carbapenemase (KPC) class A has been found worldwide, although it emerged in the eastern United States in the mid 1990s and subsequently has successfully established itself in multiple states (*1*).

Recently, a new class B enzyme, New Delhi metallo-β-lactamase (NDM-1), was characterized from a *K. pneumoniae* isolate from Sweden; the bacteria seem to have been imported from India (*2*). *Enterobacteriaceae* isolates harboring NDM-1 have now been found in multiple areas of India and Pakistan and in the United Kingdom (*3**–**5*); such isolates were recently reported from 3 US states (*6*). In Canada, carbapenemase-harboring isolates are rare, reported only for a small outbreak of clonal *Pseudomonas aeruginosa* isolates harboring IMP-7, *P. aeruginosa* isolates harboring VIM-2, a *Serratia marcescens* isolate harboring a SME-2 class A β-lactamase, and 3 isolates of *K. pneumoniae* harboring KPC-3 (*7**–**10*). We characterized NDM-harboring clinical isolates from a patient who had recently traveled to India.

## The Study

A 76-year-old woman returned to Vancouver, Canada, in early 2010 after spending 3.5 months in northern India. Before her trip, she had been in good health. In India, persistent nonbloody diarrhea developed, for which she did not seek medical attention. One month later, she was treated in the hospital for hypertension and congestive heart failure. She was discharged 3 days after admission but readmitted 3 days later because of ongoing diarrhea and decreased consciousness. Unspecified encephalitis and a urinary tract infection were diagnosed, but despite antibacterial drug therapy, her neurologic status did not improve over the next 3 weeks. She was discharged from the hospital in India and transferred to Canada.

When she arrived at the hospital in Vancouver on February 14, 2010, her vital signs reflected distributive shock: temperature 38.3°C, blood pressure 100/80 mm Hg, and heart rate 126 beats/minute. Sepsis was suspected and she was given imipenem and vancomycin. Within 24 hours, her level of consciousness had deteriorated, and she was admitted to the intensive care unit and intubated for airway protection. Blood cultures were negative, but urine culture (>1 × 10^8^ CFU/mL) grew highly drug-resistant *K. pneumoniae* N10–0469 (February 15, 2010) with intermediate resistance to chloramphenicol and susceptibility to colistin. The urine was packed with leukocytes, and no other source for sepsis was found. A perirectal sample, screened for resistant gram-negative rods, grew *K. pneumoniae* N10–0506 and *E. coli* N10–0505 (February 16, 2010). A stool specimen was negative for ova and parasites but positive for *Clostridium difficile* toxin.

The patient was given vancomycin and metronidazole for the *C. difficile* infection and colistin for the *K. pneumoniae* infection. Treatment with colistin was discontinued shortly after its initiation because of the onset of renal complications; treatment with chloramphenicol was successful. Test results for infectious causes of encephalitis (e.g., malaria smears, cerebrospinal fluid culture for bacteria, and staining for acid-fast bacteria and fungi) were negative, as were test results for cryptococcal antigen, herpes simplex viruses 1 and 2, Japanese encephalitis virus, and rabies virus. Results of computed tomographic scans, magnetic resonance imaging of the head, and an electroencephalogram were suggestive of global metabolic encephalopathy. Neurologic symptoms did not improve, despite the successful treatment of the urinary tract infection. Subsequently, *E. coli* N10–0705 isolate with an extended-spectrum β-lactamase phenotype was obtained from urine on March 9, 2010, for which the patient was treated with imipenem.

Ventilator and vasopressor support were eventually removed, and the patient was transferred to the general medicine ward. Several days later, her condition worsened and she died; the final diagnosis was toxic metabolic leukoencephalopathy, probably related to sepsis. The patient’s family refused to allow an autopsy.

**Figure 1:**
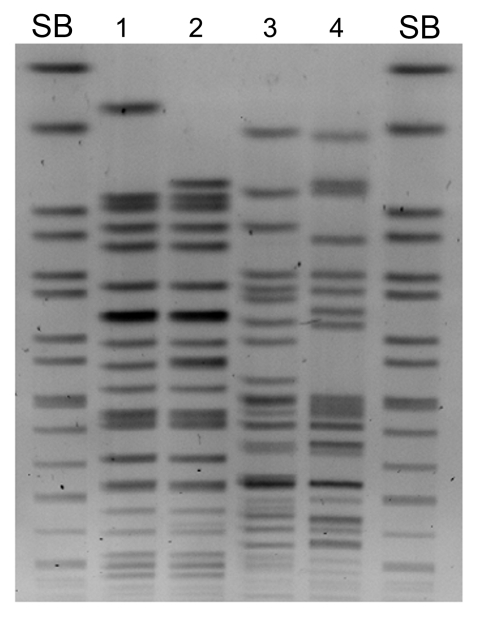
Macrorestriction analysis (*Xba*I) by pulsed-field gel electrophoresis of *Klebsiella pneumoniae* N100469 (lane 1), *K. pneumoniae* N10–0506 (lane 2), *Escherichia coli* N10–0505 (lane 3), *E. coli* N10–0705 (lane 4), *Salmonella enterica* serovar Branderup molecular mass marker (*Xba*I) (lanes SB)..

Macrorestriction analysis of the 4 isolates showed that *K. pneumoniae* N10–0469 and N10–0506 were closely related, although *E. coli* N10–0505 and N10–0705 were not (Figure 1). All 4 isolates contained multiple plasmids (data not shown). β-lactamase PCR and sequencing were conducted (primers listed in Table 1). *K. pneumoniae* N10–0469 and N10–0506 harbored the genes for SHV-1, CTX-M-15, OXA-1, CMY-6, and NDM-1; *E. coli* N10–0505 harbored TEM-1, CTX-M-15, CMY-6, and NDM-1; and *E. coli* N10–0705 harbored TEM-1, CTX-M-15, OXA-1, and CMY-42. The first characterized isolate from Sweden that harbored *bla*_NDM-1_ also harbored CMY-4, a Trp221Arg variant of the widespread CMY-2 β-lactamase (*3*). CMY-6 is a Trp221Leu variant of CMY-2 (GenBank accession no. AJ011293). CMY-42 is a Val231Ser variant of CMY-2.

**Table 1:** Primers used in study of New Delhi metallo-β-lactamase in *Klebsiella pneumoniae* and *Escherichia coli*, Canada, 2010

Name	Sequence, 5′ → 3′*	Product size, bp
SHV-UP	CGCCGGGTTATTCTTATTTGTCGC	1,016
SHV-LO	TCTTTCCGATGCCGCCGCCAGTCA	
TEM-G	TTGCTCACCCAGAAACGCTGGTG	708
TEM-H	TACGATACGGGAGGGCTTACC	
CTX-U1	ATGTGCAGYACCAGTAARGTKATGGC	593
CTX-U2	TGGGTRAARTARGTSACCAGAAYCAGCGG	
OXA1-F	CGCAAATGGCACCAGATTCAAC	464
OXA1-R	TCCTGCACCAGTTTTCCCATACAG	
CMY2-A	TGATGCAGGAGCAGGCTATTCC	323
CMY2-B	CTAACGTCATCGGGGATCTGC	
KPC1	ATGTCACTGTATCGCCGTC	863
KPC2	AATCCCTCGAGCGCGAGT	
VIM1	GTTTGGTCGCATATCGCAAC	382
VIM2	AATGCGCAGCACCAGGATAGAA	
IMP1	CCWGATTTAAAAATYGARAAGCTTG	522
IMP2	TGGCCAHGCTTCWAHATTTGCRTC	
NDM-F	GGTGCATGCCCGGTGAAATC	660
NDM-R	ATGCTGGCCTTGGGGAACG	
NDM-A	CACCTCATGTTTGAATTCGCC	984
NDM-B	CTCTGTCACATCGAAATCGC	

*H is A, C, or T; K is G or T; R is A or G; S is G or C; and W is A or T.

Antimicrobial drug susceptibility testing by Sensititer panels ESB1F and CMV1AGNF (Trek Diagnostic Systems, Cleveland, OH, USA) found that all 4 isolates were multidrug resistant and that 3 of the 4 isolates, not *E. coli* N10–0705, were nonsusceptible to carbapenems (Table 2). Nonsusceptibility to carbapenems was also shown by Etest (bioMérieux, St. Laurent, Quebec, Canada) and disk diffusion (Table 2). *K. pneumoniae* N10–0469 and *E. coli* N10–0505 were susceptible to colistin, polymixin B, and tigecycline.

**Table 2:** Antimicrobial drug susceptibilities of the isolates from study of New Delhi metallo-β-lactamase in *Klebsiella pneumoniae* and *Escherichia coli*, Canada, 2010*

Test/antimicrobial agent	*K. pneumoniae* N10–0469	*E. coli* DH10B (pNDM-Kp10469)	*E. coli* N10–0505	*E. coli* DH10B (pNDM-Ec10505)	*E. coli* DH10B
Broth microdilution (MIC, μg/mL)					
Amoxicillin/clavulanic acid	>32	>32	32	>32	4
Piercillin/tazobactam	>64	>64	>64	>64	<4
Ampicillin	>16	>16	>16	>16	2
Cefazolin	>16	>16	>16	>16	<8
Cefotaxime	>64	>64	>64	>64	<0.25
Cefotaxime/clavulanic acid	>64	>64	>64	>64	<0.12
Ceftazidime	>128	>128	>128	>128	<0.25
Ceftazidime/clavulanic acid	>128	>128	>128	>128	0.25
Cefepime	>16	16	>16	16	<1
Cefoxitin	>64	>64	>64	>64	8
Imipenem	>16	4	2	2	<0.5
Meropenem	>8	2	8	2	<1
Chloramphenicol	16	4	32	4	<2
Tetracycline	>32	≤4	32	≤4	<4
Amikacin	>64	>64	>64	>64	2
Gentamicin	>16	>16	>16	>16	<0.25
Ciprofloxacin	>2	<0.015	>2	<0.015	<0.015
Trimethoprim/sulfamethoxazole	>4	<0.12	>4	<0.12	<0.12
Etest (MIC, μg/mL)					
Aztreonam	>256	4	>256	4	0.094
Imipenem	>32	4	>32	4	0.19
Meropenem	>32	8	32	6	0.032
Ertapenem	>32	16	>32	16	0.008
Colistin	0.38	ND	0.38	ND	ND
Polymyxin B	0.19	ND	0.25	ND	ND
Tigecycline	1.5	ND	0.125	ND	ND
Disk diffusion (zone diameter, mm)					
Imipenem	6	14	11	13	33
Meropenem	6	15	10	14	35
Ertapenem	6	13	6	13	35

*NDM, New Delhi metallo-β-lactamase; ND, not done.

Multilocus sequence typing of *K. pneumoniae* N10–0469 showed that it was sequence type (ST) 16 (*11*). The first characterized strain of *K. pneumoniae* that harbored NDM-1 was ST14, unrelated to ST16 (*3*). Mutilocus sequence typing of *E. coli* N10–0505 showed it to be ST405 (*12*). The worldwide spread of *bla*_CTX-M-15_ has been partly attributed to 2 epidemic strains, ST131 and ST405 (*13*).

**Figure 2:**
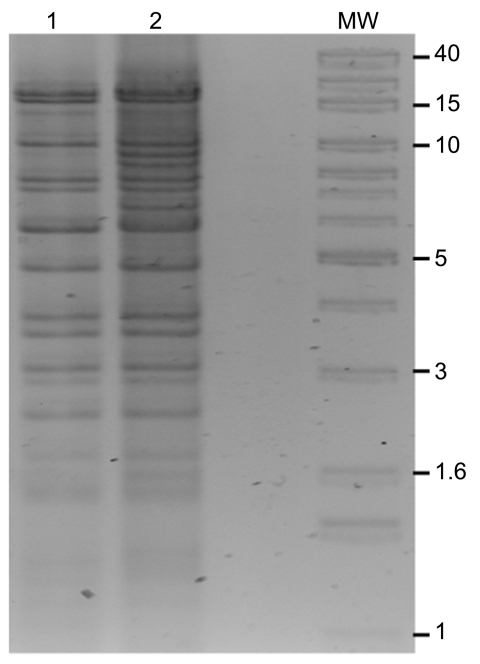
*Bgl*II restriction digests of New Delhi metallo-β-lactamase (pNDM)–Kp10469 (lane 1) and pNDM-Ec10505 (lane 2). MW, 1-kb extension ladder molecular mass marker (Invitrogen, Carlsbad, CA, USA). Sizes (kb) are indicated for some bands.

*E. coli* DH10B harboring NDM-1 plasmids was obtained by electrotransformation with whole plasmid DNA with selection on 0.25 μg/mL meropenem. Agarose gel analysis of transformants showed that they carried only a single, large plasmid. PCR and sequencing showed that CMY-6 and NDM-1 were located on an ≈102-kb plasmid (pNDM-Kp10469), originating from *K. pneumoniae* N10–0469, and on an ≈129-kb plasmid (pNDM-Ec10505), originating from *E. coli* N10–0505 (Figure 2). Incompatibility (Inc) group PCR (*14*) and fingerprinting (*15*) showed that both plasmids were related Inc A/C types, indicating possible horizontal transfer in vivo (Figure 2).

Transformant susceptibility to aztreonam is consistent with the inability of class B enzymes to efficiently hydrolyze this drug. Transformants exhibited lower carbapenem MICs than did clinical isolates, likely reflecting total β-lactamase content and additional resistance mechanisms such as porin mutations in the clinical isolates (Table 2). The recent emergence of *bla*_NDM-1_ in India has been linked to its spread on the Inc A/C-type, IncF1/FII-type, or unknown plasmid types (*3*). Analysis for class 1 integron cassettes found that both plasmids contained a 0.8-kb cassette that coded for *aac(6′)-Ib*–type aminoglycoside 6′-N-acetyl transferase. PCR for the ISCR1 element associated with some class 1 integrons was negative. Plasmids harboring NDM-1 plasmids were successfully transferred by conjugation from *K. pneumoniae* N10–0469 and *E. coli* N10–0505 to recipient strain *E. coli* RG192 (rifampin resistant).

## Conclusions

North America has thus far escaped the widespread establishment of metallo-β-lactamase–harboring organisms. Therefore, the emergence of *bla*_NDM-1_-harboring *Enterobacteriaceae* in North America is of concern because such isolates exhibit resistance to drugs commonly used to treat gram-negative infections (β-lactams, fluoroquinolones, and aminoglycosides) and have shown a propensity to spread rapidly (*4**–**6****)***)**.**
